# “Hangry” in Forensic Psychiatry? Analysis of the Relationship Between Eating Disorders and Aggressive Behavior in Patients with Substance Use Disorders

**DOI:** 10.3390/brainsci15080836

**Published:** 2025-08-04

**Authors:** Judith Streb, Tinatin Deisenhofer, Samira Schneider, Victoria Peters, Manuela Dudeck

**Affiliations:** Department of Forensic Psychiatry and Psychotherapy, Ulm University, 89312 Günzburg, Germany; tinatin-deisenhofer@outlook.de (T.D.); samira.aishat.schneider@gmail.com (S.S.); manuela.dudeck@bkh-guenzburg.de (M.D.)

**Keywords:** forensic psychiatry, eating disorders, substance use disorder, aggression, excitability, spontaneous aggression, reactive aggression, violent offense

## Abstract

**Background/Objectives**: Substance use disorders and eating disorders frequently co-occur and are both associated with increased aggression. As a result, individuals with these conditions are overrepresented in prison populations. The present study investigated whether symptoms of eating disorders in male forensic psychiatric inpatients with substance use disorders are associated with heightened aggression. To this end, various forms of aggressive behavior—including spontaneous and reactive aggression, excitability, and violent offenses—were analyzed. **Methods**: Fifty-six male patients from two forensic psychiatric hospitals in Germany participated in the study. Symptoms of eating disorders were evaluated with the German version of the Eating Disorder Examination Questionnaire (EDE-Q), and aggression was measured with the Short Questionnaire for the Assessment of Aggression Factors (K-FAF) and by considering the violent index offense. Data were analyzed by generalized linear models, with age and body mass index (BMI) included as covariates. **Results**: Higher EDE-Q scores significantly predicted increased spontaneous aggression and excitability. However, no significant association was found between eating disorder symptoms and reactive aggression or the likelihood of a violent index offense. Age and BMI did not significantly influence any aggression subscales. **Conclusions**: The findings suggest that in patients with substance use disorder, eating disorder symptoms may be linked to heightened internalized forms of aggression. These results support the clinical relevance of screening for eating disorder symptoms in forensic psychiatric settings and integrating dietary interventions into therapeutic efforts to manage aggression.

## 1. Introduction

A growing body of clinical research highlights a significant overlap between addiction and eating disorders, and several studies have documented high rates of disordered eating behaviors among individuals struggling with substance abuse. For instance, Peveler and Fairburn [1] found that 36% of alcohol-dependent patients undergoing withdrawal therapy exhibited binge-eating symptoms and 26% met the criteria for a clinically acute eating disorder. Notably, 19% reported a prior diagnosis of anorexia nervosa. Similarly, Jonas et al. [2] identified eating disorders—specifically anorexia and bulimia—in 32% of individuals who contacted a national cocaine helpline for drug-related concerns. In another study, Lacey and Moureli [3] observed that 40% of patients with alcohol-related problems engaged in regular binge-eating episodes. Overall, the reported prevalence of eating disorders among individuals with substance use disorders ranges from 19% to 40%, underscoring the need for integrated approaches to diagnosis and treatment.

Eating disorders can significantly impact the lives of those affected and cause considerable psychological distress [4]. The primary forms of eating disorders include anorexia nervosa, bulimia nervosa, and binge-eating disorder. Anorexia nervosa is characterized by restrictive eating behavior and significantly reduced body weight [5], and bulimia nervosa is marked by episodes of excessive food intake followed by compensatory behaviors such as vomiting, the use of laxatives, or excessive exercise, depending on the subtype of the disorder. In contrast, binge-eating disorder involves episodes of excessive food consumption without any compensatory measures, so it can lead to secondary conditions such as obesity [5]. The prevalence of anorexia nervosa and bulimia nervosa is significantly higher in women than in men [6]. Thus, the female-to-male ratio is approximately 10:1 for anorexia nervosa, 20:1 for bulimia nervosa, and 1.5:1 for binge-eating disorder [5], reinforcing the common perception of eating disorders being more frequent in women.

In the clinical context, individuals with eating disorders—particularly bulimia nervosa—tend to exhibit aggressive behavior more frequently [7,8,9]. Aggression, in turn, affects the course of eating disorders and treatment outcomes [8,9,10,11,12]. Furthermore, a study by Swami et al. [13] showed that higher levels of self-reported hunger are associated with stronger feelings of irritability and anger (note that anger is an emotional reaction, whereas aggression is a destructive behavior that often results from anger). This connection is illustrated by the colloquial English term “hangry,” which captures the idea that people tend to feel angry when they are hungry.

Given these links between eating pathology and aggression, it is particularly noteworthy that individuals with eating disorders are significantly overrepresented in prison populations [14,15]. A study from the British prison system estimated the prevalence of eating disorders among male inmates at 15.1% [16], which is significantly higher than the lifetime prevalence among men in the general population of 2.2% (range, 0.8 to 6.5% [17]). Affected individuals are assumed to use eating disorders as a means of countering the powerlessness experienced in the prison context and to thereby seek to regain a sense of control, at least over their own bodily functions. Similarly, mentally ill individuals who are permanently housed in secure psychiatric facilities and who report having gained significant weight—often involuntarily, from their perspective—cite feelings of helplessness and despair as the cause. They also report being unable to maintain a healthy diet according to their own preferences, in part because of food access restrictions and rigid meal schedules [18]. Individuals struggling with addiction often exhibit aggressive behavior in closed environments [19]. This can stem from frustration, withdrawal symptoms, or a sense of confinement, exacerbating their already challenging emotional state. Addressing these behaviors requires a supportive approach that considers both the underlying addiction and the immediate triggers contributing to their aggression.

Therefore, the present study aimed to investigate whether eating disorders in incarcerated offenders with substance-use disorders are associated with feelings of anger or various forms of aggressive behavior, including spontaneous and reactive aggression and excitability. Additionally, the study examined whether the index offense of forensic psychiatric patients with an eating disorder was more frequently associated with violence. The presence of an eating disorder was assessed with the German version of the Eating Disorder Examination Questionnaire [20], and anger and aggressive behavior were measured with the Short Questionnaire for the Assessment of Aggression Factors (K-FAF, [21]).

Understanding the interaction between eating pathology and aggression has significant implications, particularly for forensic and psychiatric public health. Recognizing how disordered eating behaviors can contribute to or be influenced by aggressive tendencies may lead to more comprehensive treatment approaches in these settings. In forensic populations, where aggression is often a key concern, addressing underlying eating disorders could help reduce violent behavior and improve overall mental health outcomes. Furthermore, in psychiatric public health, integrating strategies that consider both eating pathology and aggression could enhance prevention and intervention programs, offering more holistic care for individuals at risk.

## 2. Materials and Methods

### 2.1. Procedures

Data were collected from 2021 to 2023. Study participants were recruited by directly approaching patients on wards in the forensic psychiatric departments of the district hospitals in Günzburg and Kaufbeuren, Germany. If they expressed interest in participating, patients were taken either individually or in small groups to a separate room, where they were given written information on the study and orally briefed about the procedures, the voluntary nature of participation, and data protection measures. All participants signed an informed consent form. The exclusion criterion was a lack of proficiency in the German language. Then, height and weight were measured, and participants were asked to complete the questionnaires anonymously and then submit them in an envelope to the study management team. The study was performed in accordance with the Declaration of Helsinki and approved by the ethics review board of Ulm University (No. 346/20).

### 2.2. Sample

Fifty-six male patients from the departments of forensic psychiatry and psychotherapy at the district hospitals in Günzburg (*n* = 24) and Kaufbeuren (*n* = 32) participated in this study. The participants had been convicted according to Section 64 of the German Criminal Code, i.e., for crimes in the context of substance-related diagnoses; the diagnoses corresponded to the DSM-V concept of substance use disorders [22]. Under German criminal law, a court can order placement in a forensic psychiatric hospital if there is a risk that as a result of a substance use disorder a person is likely to commit significant unlawful acts again.

### 2.3. Materials

#### 2.3.1. German Version of the Eating Disorder Examination Questionnaire

The Eating Disorder Examination Questionnaire (EDE-Q) [20,23] is a tool for measuring the characteristic psychopathology of eating disorders in adults and adolescents. It includes four subscales: restraint (example item: “Have you gone for long periods of time (8 waking hours or more) without eating anything at all in order to influence your shape or weight?”), eating concern (example item: “Have you had a definite fear of losing control over eating?”), weight concern (example item: “Has your weight influenced how you think about (judge) yourself as a person?”), and shape concern (example item: “Have you had a definite desire to have a totally flat stomach?”). The extent of these characteristics within the past 28 days is assessed with 22 items, and participants rate the frequency or intensity of the traits on 7-point Likert scales (“no days” to “every day” or “not at all” to “markedly,” respectively). The EDE-Q is evaluated by calculating the mean scores for the subscales and an overall score. Empirical studies have confirmed the reliability (Cronbach’s alpha is 0.78 and 0.89 for the subscales and 0.94 for the overall score [24]) and validity [20] of the EDE-Q, and reference values are available for various populations [24].

#### 2.3.2. Short Questionnaire for the Assessment of Aggression Factors

The German Short Questionnaire for the Assessment of Aggression Factors (K-FAF, [21]) measures aggressive behavior by using 49 items that are rated on a 6-point Likert scale ranging from 0 (“does not apply at all”) to 5 (“fully applies”). The K-FAF, a revised short version of the Questionnaire for the Assessment of Aggression Factors, identifies aggression factors based on five subscales: spontaneous aggression, reactive aggression, excitability, self-aggression, and inhibition of aggressive behavior. The present study focused on the subscales spontaneous aggression, reactive aggression, and irritability. The spontaneous aggression subscale (example item: “Sometimes I enjoy torturing others”) refers to imagined, verbal, or physical aggression against people and animals and includes sadistic tendencies; individuals with high scores are impulsive, and typically, men score higher than women. Reactive aggression (example item: “When someone treats me badly, I want revenge”) is a socially widely accepted form of aggression; high scores indicate a strong drive to assert oneself with a conformist mindset, whereas low scores suggest a rejection of an aggressive behavior style. Excitability (example item: “I often raise my voice during an argument”) describes affect regulation, and high scores indicate frequent experiences of anger, rage, and fury and low frustration tolerance. The authors report good internal consistency of the subscales (Cronbach’s alpha values: spontaneous aggression, 0.77; reactive aggression, 0.77; and excitability, 0.84) and confirm the validity of the K-FAF, i.e., its ability to significantly differentiate between violent offenders and non-offenders [21].

### 2.4. Data Analysis

Data were analyzed with IBM SPSS Statistics for Windows, Version 26 (Armonk, NY, USA: IBM Corp.). Descriptive statistics (mean, SD, and absolute and relative frequencies) were calculated. To examine how eating disorders (measured by the EDE-Q) influenced aggressive behavior (measured by the K-FAF), generalized linear models (GZLM) with normal distribution and identity link function were computed. The dependent variables were the three K-FAF subscales, i.e., spontaneous aggression, reactive aggression, and excitability. The potentially confounding variables age and BMI were included in the model as covariates. The relationship between the presence of an eating disorder and a violent offense as the index offense was analyzed with a GZLM with binomial distribution and logit link function. The dependent variable was the binary variable “violent offense” (0 = no violent offense; 1 = violent offense), and the predictors included in the model were the EDE-Q total score, age, and BMI. The classical linear model (with linear regression and analysis of variance as important special cases) is suitable only for analyzing metric outcome variables and assumes uncorrelated, homoscedastic, and normally distributed residuals. These assumptions were violated, so it was advisable to use a GZLM. In contrast to classical models, a GZLM does not model the expected value of the outcome variable directly, but rather the result of a transformation applied to this expected value. For the GZLM analyses, missing values were handled using listwise deletion, meaning that only cases with complete data on all included variables were analyzed.

## 3. Results

Sociodemographic and forensic psychiatric characteristics of the patient sample can be found in Table 1.

The mean (SD) height of the sample was 1.78 (0.07) meters, and the mean (SD) weight was 89.13 (14.44) kg (range, 66.10–126.90 kg). The mean (SD) body mass index (BMI), which relates body weight to height, was 28.07 (4.34), indicating a tendency towards overweight in the sample (see [25]).

The results of the GZLM on the factors that predicted the score on the K-FAF Spontaneous Aggression subscale are presented in Table 2. As shown in the table, the EDE-Q total score proved to be a significant positive predictor, and an increase of 1 point in the EDE-Q total score led to an increase of 3.021 points in the score on the spontaneous aggression subscale. Neither age nor BMI were significantly associated with the spontaneous aggression score.

In the GZLM for the K-FAF reactive aggression subscale, none of the predictors, i.e., age, BMI, and EDE-Q total score, proved to be significant in predicting the total score (see Table 3).

Table 4 shows the results of the GZLM on whether age, BMI, or EDE-Q total score predicted the score on the K-FAF excitability subscale. Neither age nor BMI were significant predictors; however, the EDE-Q total score significantly predicted the excitability subscale score, and an increase of 1 point in the EDE-Q total mean score led to an increase of 5.720 points in the excitability score, i.e., the more disordered the eating behavior, the higher the excitability score.

The GZLM on factors that predicted whether or not the index offense was violent (0 = no, 1 = yes) is shown in Table 5. None of the three predictors, i.e., age, BMI, and EDE-Q total score, proved to be significant.

## 4. Discussion

This study found that the EDE-Q total score appears to play a significant role in predicting spontaneous aggression and excitability in forensic patients, but that it does not have significant effects on reactive aggression or the likelihood that the index offense was violent. These findings can be explained by considering how the construct of aggression is measured: Classical models of human aggression—both psychological and biological—distinguish between two fundamentally different types of aggression, i.e., reactive aggression, which occurs in response to an acute threat (as a form of protection against threatening stimuli) and spontaneous (also called instrumental or appetitive) aggression, which is goal-directed and internally motivated and involves harmful behavior toward others to obtain positive reinforcement [26]. According to classical learning theory, reactive aggression is modulated by negative reinforcement (i.e., the termination of an aversive state), whereas spontaneous aggression is triggered by positive reinforcement (e.g., the pleasure derived from aggressive behavior). These definitions explain why individuals with an eating disorder score higher on the EDE-Q spontaneous aggression and excitability subscales, but not on the reactive aggression subscale, i.e., this difference is related to the fact that reactive aggressive behavior usually only occurs in emergency situations where one is forced to defend oneself, so it is not internally motivated and therefore not related to one’s level of satiety.

The study also found no connection between the presence of an eating disorder and a violent offense as the index offense. A possible reason for this lack of a significant association may be that violent offenses are always the result of multiple factors (such as social status, substance use disorders, and a criminal environment). Furthermore, we did not collect data on whether the eating disorder existed prior to placement in forensic psychiatric care. If the eating disorder only manifested during the stay in forensic care, then it would be unrelated to the initial offense, which occurred earlier.

The link between substance use and aggressive behavior is complex and not solely causal. Emerging evidence also points to the role of nutrition in modulating aggression in that eating disorders are associated with heightened spontaneous aggression and excitability. These findings emphasize the multifaceted biopsychosocial factors that contribute to violent behavior.

The study has some limitations. First, it was a correlational study, so no conclusions can be drawn regarding causal relationships between variables. Second, violent offenses were predicted statistically, although the data on index offenses were retrospective. Future violent offenses would have served as a more accurate indicator, but such data were not available. Third, aggression was measured by self-report, which introduces the risk of socially desirable response bias. Particularly aggressive offenders may underreport their aggressive behavior to appear more favorable or to avoid negative consequences such as legal repercussions, stricter supervision, or delayed release [27]. Additionally, some individuals may lack insight into their own behavior or misinterpret questions due to cognitive distortions, denial, or personality traits commonly linked to antisocial or aggressive tendencies. Fourth, the relatively small sample size may limit the generalizability of the findings. With fewer participants, the results might not accurately represent the broader population, increasing the risk that observed effects are due to random variation rather than true associations. To address this concern, we conducted a post hoc power analysis. Since G*Power does not support GZLM, we used multiple linear regression as a comparable test. Assuming a medium effect size (f^2^ = 0.15), a significance level of 5%, and a power of 0.8, the required sample size is 55. As our actual sample size was 56, it meets the power analysis requirements. Fifth, data were collected between 2021 and 2023, during the post-COVID-19 recovery and adaptation period. This timing may have influenced participants’ behavior and overall responses. These factors should be considered when interpreting the results and comparing. Last, other important variables—such as psychiatric comorbidity, medication use, length of stay, and prior traumatic experiences—may also influence aggressive behavior and should be considered as potential confounders. These factors can significantly affect an individual’s emotional regulation, stress response, and behavior in closed settings. For example, untreated psychiatric disorders or side effects of certain medications may exacerbate aggression, while longer stays or a history of trauma can impact how individuals cope with confinement.

## 5. Conclusions

The findings of this study have implications for clinical practice. Eating disorders are often not routinely diagnosed in forensic psychiatric settings because they are typically assumed to have no relevance to the criminal offense. However, the present study suggests that a substantial proportion of patients with substance use disorders also exhibit symptoms of an eating disorder. Therefore, we recommend that these symptoms be systematically assessed and addressed at the time of admission to forensic psychiatric facilities. Psychiatric care protocols in forensic settings should implement standardized tools to identify symptoms of disordered eating (as part of aggression risk assessments). Including eating pathology in routine assessments would allow for more precise case formulations and treatment plans. Promoting healthy eating patterns and behaviors may help to reduce excitability, anger, and aggression, and future research should further explore other potential benefits.

Future research should aim to build on these findings by incorporating more objective measures and expanding the scope of investigation. One important direction is the exploration of biological markers, which may be linked to both eating pathology and aggression. These biomarkers could help clarify underlying mechanisms and improve the precision of risk assessments. Additionally, future studies should include behavioral assessments of aggression—such as staff observations, incident reports, or structured behavioral tasks—to complement self-reported data. This multimethod approach would reduce bias and offer a more comprehensive understanding of how eating disorders may relate to aggressive behavior in forensic populations.

## Figures and Tables

**Table 1:** Sociodemographic and forensic psychiatric characteristics of the patient sample (all of whom were detained under Section 64 of the German Criminal Code at the Departments of Forensic Psychiatry and Psychotherapy at the District Hospitals in Günzburg and Kaufbeuren; data collection period: 2021–2023).

	Sample (N = 56) M (SD)/n (%)
Age, y ^1^	34.51 (9.02)
Highest level of education	
Did not complete school	4 (7%)
Graduated after 9 years	17 (30%)
Graduated after 10 years	34 (61%)
Graduated from high school (i.e., after 12 or 13 years)	7 (13%)
Marital status	
Married/in a committed partnership	17 (30%)
Single	31 (55%)
Divorced	6 (11%)
Widowed	2 (4%)
Primary diagnosis (ICD-10) ^1^	
F10: Alcohol-related disorder	9 (16%)
F11: Opioid-related disorder	3 (5%)
F12: Cannabinoid-related disorders	5 (9%)
F14: Cocaine-related disorder	13 (23%)
F15: Other stimulant-related disorder	2 (4%)
F19: Multiple substance-use disorders	23 (42%)
Secondary diagnosis of a personality disorder (ICD-10)	
F60.2: Dissocial	5 (9%)
Age at first illness onset, y ^2^	17.23 (7.89)
Number of previous inpatient psychiatric stays ^3^	1.45 (3.50)
Duration of current detention, mo ^4^	14.11 (10.24)
Most serious index offense ^1^	
(Attempted) murder/manslaughter	1 (2%)
Robbery	1 (2%)
Assault	9 (16%)
Property offense	6 (11%)
Violation of the Weapons Act	3 (5%)
Violation of the Narcotics Act	35 (63%)
Age at index offense, y ^4^	21.06 (8.80)
Number of previous convictions ^5^	6.43 (6.11)

Notes: N, sample size; M, mean; n, absolute number; %, frequency in percent; ICD-10, International Classification of Mental Disorders. ^1^ Missing values = 1; ^2^ missing values = 9; ^3^ missing values = 7; ^4^ missing values = 4; ^5^ missing values = 2.

**Table 2:** Results of the generalized linear model predicting the score on the spontaneous aggression subscale of the Short Questionnaire for the Assessment of Aggression Factors (K-FAF) in a forensic patient sample (N = 56 ^1^).

	*b*	SE (*b*)	Wald-Chi^2^	Significance
Age	−0.060	0.122	0.247	0.619
BMI	−0.003	0.285	0.000	0.990
EDE-Q total score	3.021	1.274	5.618	0.018

Notes: ^1^ Missing values = 3; BMI, body mass index; EDE-Q, Eating Disorder Examination Questionnaire.

**Table 3:** Results of the generalized linear model predicting the score on the reactive aggression subscale of the Short Questionnaire for the Assessment of Aggression Factors (K-FAF) in a forensic patient sample (N = 56 ^1^).

	***b***	SE (*b*)	Wald-Chi^2^	Significance
Age	−0.219	0.176	1.538	0.215
BMI	−0.134	0.414	0.104	0.747
EDE-Q total score	3.302	1.858	3.160	0.075

Notes: ^1^ Missing values = 4; BMI, body mass index; EDE-Q, Eating Disorder Examination Questionnaire.

**Table 4:** Results of the generalized linear model predicting the score on the excitability subscale of the Short Questionnaire for the Assessment of Aggression Factors (K-FAF) in a forensic patient sample (N = 56 ^1^).

	*b*	SE (*b*)	Wald-Chi^2^	Significance
Age	0.160	0.169	0.901	0.342
BMI	−0.455	0.395	1.326	0.249
EDE-Q total score	5.720	1.766	10.491	0.001

Notes: ^1^ Missing values = 3; BMI, body mass index; EDE-Q, Eating Disorder Examination Questionnaire.

**Table 5:** Results of the generalized linear model on factors that predicted whether or not the index offense was violent in a forensic patient sample (N = 56 ^1^).

	*b*	SE (*b*)	Odds Ratio	Significance
Age	−0.015	0.035	0.985	0.668
BMI	−0.093	0.090	0.911	0.303
EDE-Q total score	0.503	0.347	1.654	0.147

Notes: ^1^ Missing values = 2; BMI, body mass index; EDE-Q, Eating Disorder Examination Questionnaire.

## Data Availability

The raw data supporting the conclusions of this manuscript will be made available by the authors, without undue reservation, to any qualified researcher. Requests to access the datasets should be directed to judith.streb@uni-ulm.de.

## Abbreviations

The following abbreviations are used in this manuscript:

StGB	German criminal code
N	Sample size
M	Mean
SD	Standard deviation
n	Absolute number
ICD-10	International Classification of Mental Disorders
EDE-Q	Eating Disorder Examination Questionnaire
K-FAF	Short Questionnaire for the Assessment of Aggression Factors
BMI	Body mass Index
b	Unstandardized regression coefficient
GZLM	Generalized linear models
